# Strategies for initial management of hypertension

**Published:** 2010-11

**Authors:** Rajeev Gupta, Soneil Guptha

**Affiliations:** Department of Medicine, Fortis-Escorts Hospital, Jaipur, India; * *Regional Headquaters, MSD Technology Singapore Pte Ltd, Singapore*

**Keywords:** B-blockers, dietary therapy, high blood pressure, lifestyle, pharmacotherapy, physical activity, salt

## Abstract

High blood pressure (BP) is a major public health problem in India and its prevalence is rapidly increasing among urban and rural populations. Reducing systolic and diastolic BP can decrease cardiovascular risk and this can be achieved by non-pharmacological (lifestyle measures) as well as pharmacological means. Lifestyle changes should be the initial approach to hypertension management and include dietary interventions (reducing salt, increasing potassium, alcohol avoidance, and multifactorial diet control), weight reduction, tobacco cessation, physical exercise, and stress management. A number of pharmaceutical agents, well evidenced by large randomized clinical trials, are available for initial treatment of high BP. These include older molecules such as thiazide diuretics and beta-blocking agents and newer molecules, dihydropyridine calcium channel blockers (CCB), angiotensin converting enzyme (ACE) inhibitors, and angiotensin receptor blockers (ARB). In view of the recent clinical trials data, some international guidelines suggest that CCB, ACE inhibitors or ARB and not beta-blockers or diuretics should be the initial therapy in hypertension management. Comprehensive hypertension management focuses on reducing overall cardiovascular risk by lifestyle measures, BP lowering and lipid management and should be the preferred initial treatment approach.

## Introduction

High blood pressure (BP) is a major public health problem in India and elsewhere^1^–^4^. It is a major cardiovascular risk factor^5^–^7^ and contributes significantly to cardiovascular mortality^8^,^9^. Prospective Studies Collaboration has reported that reducing BP can substantially decrease cardiovascular risk and cardiovascular as well as all-cause mortality^10^. This risk reduction is steeper in younger subjects than in the older subjects (Fig.) and is more when baseline blood pressure levels are high. In a meta-analysis of 61 studies involving more than a million patients with hypertension and 12.7 million years of follow up it was observed that reducing systolic as well as diastolic BP reduced cardiovascular events^11^. At ages 40-69 yr, each difference of 20 mmHg systolic BP or 10 mm Hg diastolic BP was associated with more than a two-fold difference in the stroke death rate, and with two-fold differences in the death rates from coronary heart disease and other vascular causes. All of these proportional differences in vascular mortality were about half as extreme at ages 80-89 yr as at ages 40-49 yr, but the annual absolute differences in risk were greater in old age.

**Figure 1:**
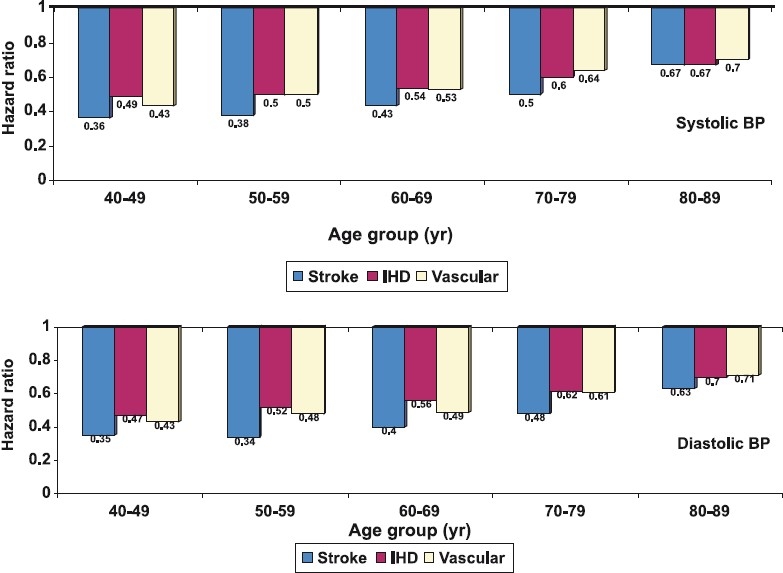
Prospective Studies Collaboration analysis on influence of high blood pressure on cardiovascular mortality. Reduction of usual systolic BP (upper panel) and diastolic BP (lower panel) is associated with a lower hazard ratios (hazard ratio <1.0) for mortality from stroke, ischaemic heart disease (IHD) as well as other vascular causes. The hazard ratios are much more in the younger age-groups indicating more benefit by BP reduction at these age-groups (*Source*: Ref^10^).

There is, therefore, a need to lower BP in all groups of patients. This can be achieved by non-pharmacological (lifestyle measures) as well as pharmacological means^12^. Lifestyle changes include dietary interventions, weight control, tobacco cessation, exercise, and stress management. A number of pharmaceutical agents, well evidenced by large randomized clinical trials, are available for initial treatment of high BP. These include older molecules such as thiazide diuretics and beta-blocking agents, and newer molecules such as dihydropyridine calcium channel blockers (CCB), angiotensin converting enzyme (ACE) inhibitors, and angiotensin receptor blockers (ARB). Comprehensive hypertension management focuses on reducing overall cardiovascular risk and should be the preferred approach for initial management of hypertension. This article focuses on initial management strategies in hypertension using non-pharmacological as well as pharmacological approaches.

## NON-PHARMACOLOGICAL MANAGEMENT

Initial management of hypertension uses a two-pronged approach, with emphasis on lifestyle measures and add-on drug management^13^. Non-pharmacological therapy (or lifestyle management) has an important role in both non-hypertensive and hypertensive individuals. In non-hypertensive individuals, including those with pre-hypertension, lifestyle modifications have the potential to prevent hypertension and more importantly to reduce BP and lower the risk of BP-related clinical complications. In hypertensive individuals, lifestyle modifications can serve as initial treatment before the start of drug therapy and as an adjunct to drug therapy in persons already on medication. In hypertensive individuals with medication-controlled BP these therapies can facilitate drug step-down in individuals who can sustain lifestyle changes.

Treatment is successful when multiple factors in the patient’s life are addressed, since essential hypertension is considered as a result of interactions between genes and environment^14^. The environmental effects are powerful and explain most of the BP differences between individuals and populations and their control in management of high BP is crucial^14^. Important lifestyle or environmental factors are dietary excess of sodium and fat, dietary deficiency of potassium and fibre, alcohol intake, physical inactivity, and psychosocial stress^13^. Obesity, especially, truncal obesity are powerful proximate determinants of high BP, also in Indians^15^, and lifestyle influences on their genesis are well known. Major lifestyle factors influencing hypertension management and amenable to control are shown in Table I.

**Table I:** Dietary and lifestyle changes that modify blood pressure

	Level of evidence	Recommendations
Dietary sodium intake	++	<100 mmol (2.3 g) of sodium per day
Dietary potassium intake	++	>120 mmol (4.7 g) of potassium per day
Omega-3 polyunsaturated fat	++	Increase omega-3 fat intake from natural sources
Overall healthy dietary patterns	++	An overall healthy diet: DASH diet (USA), Mediterranean diet (Europe), Ornish Diet (USA), Indian vegetarian diet (India)
Dietary calcium, magnesium	+/−	Increase dietary calcium and magnesium intake through natural sources
Saturated fat, omega-6 unsaturated fat, monounsaturated fat	+/− to +	Low saturated fat diet for reducing the cardiovascular risk
Protein, total protein, animal protein, vegetable protein	+/− to +	Increase vegetable protein in place of carbohydrates
Carbohydrate	+	Amount and type of carbohydrate uncertain
Fibre	+	High fibre diet
Cholesterol	+/−	Low cholesterol diet to reduce cardiovascular risk
Exercise	+	At least 30 min of moderate activity most days of the week
Alcohol intake	++	Moderation of alcohol intake to <2 drinks/day in men and <1 drink/day in women in those who take alcohol Stress management
Stress management	+/−	Yoga, meditation, progressive relaxation techniques

+/− indicates limited or equivocal evidence; + suggestive evidence, typically from observational studies and some clinical trials; ++ persuasive evidence, typically from randomized clinical trials. DASH dietary approaches to stop hypertension. *Source*: Adapted from Ref 13, 21, 59, 66 and 78

Lifestyle measures, are a crucial step in hypertension management. Dietary Approaches to Stop Hypertension (DASH) study^16^ showed that a diet low in sodium and high in fruits, vegetables, and calcium is helpful in treating hypertension. Exercise is critically important, especially in children and young adults with hypertension who often have heightened sympathetic nervous system activity. Patients with hypertension often feel stressed, and the stress aggravates their BP.

Numerous short-term trials have documented that individuals can make these lifestyle changes which lower BP^13^. A more recent trial^17^ has documented that individuals can simultaneously make multiple lifestyle changes but a vexing issue is the extent to which individuals can sustain lifestyle changes over the long term. This issue has been addressed in several long term trials such as phase 2 of the Trials Of Hypertension Prevention (TOHP2)^18^,^19^, and Trials Of Non-pharmacological interventions in the Elderly (TONE)^20^. These studies reported that in middle aged (TOHP2) as well as elderly (TONE) subjects with mild to moderate hypertension, diet, exercise induced weight loss and sodium restriction can be sustained and are associated with significant BP reductions. However, it is important to individually evaluate various factors (listed in Table I) for their importance in initial management of hypertension.

### Dietary factors

Dietary modifications are mainstay for prevention and initial treatment of hypertension^21^. In hypertensive patients, in addition to a well-balanced diet, the dietary sodium intake should be limited to 65 to 100 mmol/day. Other recommendations are: following a diet low in saturated fat and cholesterol, and one that emphasizes fruits, vegetables and low-fat dairy products, dietary and soluble fibre, whole grains and protein from plant sources^21^ Alcohol intake should be moderated.

### Reduced salt intake

Evidence from animal studies, epidemiological studies, clinical trials, and meta-analyses suggests that with increase in dietary salt (sodium chloride) intake, BP also rises. The most persuasive evidence about the effects of salt on BP comes from rigorously controlled, dose response trials^21^–^23^. Each of these trials tested sodium levels and documented significant relationships. The largest of the dose response trials, the DASH-Sodium trial^22^, tested the effects of 3 different doses of sodium intakes separately in two distinct diets: the DASH diet and a control diet (more typical of what Americans eat). BP reduction was the highest in the group with the lowest sodium levels. In addition, clinical trials have documented that a reduced sodium intake can prevent hypertension (relative risk reduction of about 20% with or without concomitant weight loss), can lower BP in the setting of antihypertensive medication, and can facilitate hypertension control^24^. In observational studies, reduced sodium intake is associated with a blunted age-related rise in systolic BP. In other observational studies, reduced salt intake is associated with a reduced risk of atherosclerotic cardiovascular events and congestive heart failure^21^.

The BP response to differences and changes in intake of dietary sodium intake is heterogeneous^25^ (as is the BP response to other dietary changes). Despite use of the terms “salt sensitive” and “salt resistant” to classify individuals in research studies, the change in BP in response to a change in salt intake is not binary. Rather, the reduction in BP from a reduced sodium intake has a continuous distribution, with individuals having greater or lesser degrees of BP reduction^26^. In general, the effects of sodium reduction on BP tend to be greater in blacks; middle-aged and older persons; and individuals with hypertension, diabetes, or chronic kidney disease. In Indian arm of the international INTERSALT study, response of Indian subjects to dietary sodium was no different from other groups^27^.

The Indian Council of Medical Research (ICMR) recommends 1.5 g/d (65 mmol/d) sodium as an adequate intake level, primarily to ensure nutrient adequacy^28^. Although a sodium intake below this level is associated with lower BP, little information is available about the nutrient content of diets that provide <1.5 g/d of sodium. From the DASH-Sodium trial, it is apparent that western-type diets can provide this level of sodium intake and that such a diet can also provide adequate levels of other nutrients^22^. Because the relationship between sodium intake and BP is direct and progressive without an apparent threshold, it is difficult to set an upper level of sodium intake, which could be 65 mmol/day (4 g salt). However, in view of the available food supply and the currently high levels of sodium consumption, a reduction in sodium intake to 65 mmol daily is not easily achievable. In the interim, a reasonable recommendation is an upper limit of 100 mmol of sodium equivalent to 6 g common salt/day. In aggregate, available data strongly support current, population-wide recommendations to lower salt intake. To reduce salt intake, people should choose foods low in salt and limit the amount of salt added to food. However, because >75 per cent of consumed salt in India comes from home cooked foods, any meaningful strategy to reduce salt intake must involve public education to change consumption^29^. In view of the escalating influence of food industry in India focus should also be on the food manufacturers and restaurants, which should progressively reduce the salt added to foods by 50 per cent.

### Increased potassium intake

High potassium intake is associated with reduced BP. Although data from individual trials have been inconsistent, three meta-analyses of these trials have documented a significant inverse relationship between potassium intake and BP in non-hypertensive and hypertensive individuals^21^. In the meta-analysis by Whelton *et al*^30^ average systolic and diastolic BP reductions associated with increase in urinary potassium excretion of 2 g/d (50 mmol/d) were 4.4 and 2.5 mm Hg in hypertensive and 1.8 and 1.0 mm Hg in non-hypertensive individuals. Available data suggest that increased potassium has beneficial effects on BP in the setting of salt intake that is low. Potassium reduces BP to a greater extent in blacks than in whites. A study from India reports similar BP reduction with potassium supplementation as observed in the Caucasian whites^31^.

Because a high potassium intake can be achieved through diet rather than pills and because potassium derived from foods is also accompanied by a variety of other nutrients, the preferred strategy to increase potassium intake is to consume foods such as fruits and vegetables rich in potassium, rather than supplements. In the DASH trial, the two groups that increased fruit and vegetable consumption both lowered BP.^22^ In the generally healthy population with normal kidney function, a potassium intake from foods >4.7 g/d (120 mmol/d) poses no risk because excess potassium is readily excreted in the urine. However, in individuals whose urinary potassium excretion is impaired, a potassium intake <4.7 g/d (120 mmol/d) is appropriate because of adverse cardiac effects from hyperkalaemia. Common drugs that can impair potassium excretion are ACE inhibitors, ARBs, nonsteroidal antiinflammatory agents, and potassium-sparing diuretics. Medical conditions associated with impaired potassium excretion include diabetes, chronic renal insufficiency, end-stage renal disease, severe heart failure, and adrenal insufficiency. Elderly individuals are at increased risk of hyperkalaemia because they often have one or more of these conditions or take one or more medications that impair potassium excretion.

### Moderation of alcohol intake

Observational studies and clinical trials have documented a direct, dose-dependent relationship between alcohol intake and BP, particularly when the intake of alcohol increases above 2 drinks per day^32^. Importantly, this relationship has been shown to be independent of potential confounders such as age, obesity, and salt intake. A recent meta-analysis of 15 randomized controlled trials reported that decreased consumption of alcohol reduced systolic and diastolic BP by 3.3 and 2.0 mm Hg, respectively^33^. BP reductions were similar in non-hypertensive and hypertensive individuals. Available evidence supports moderation of alcohol intake (among those who drink) as an effective approach to lower BP. Alcohol consumption should be limited to ≤ 2 alcoholic drinks per day in most men and ≤1 alcoholic drink per day in women and lighter-weight persons. Note that one drink is defined as 360 ml of regular beer, 150 ml of wine (12% alcohol), and 45 ml of 80-proof distilled spirits.

### Increase in fibre, fruits and vegetables

Dietary fibre consists of the indigestible components of food from plants. Evidence from observational studies and several clinical trials suggests that increased fibre intake may reduce BP^13^. More than 40 randomized trials of dietary fibre supplementation have been conducted^21^. Still, most did not have BP as their primary outcome, and many had a multicomponent intervention. A meta-analysis of these trials, restricted to the 20 trials that increased just fibre intake, documented that supplemental fibre (average increase, 14 g/d) was associated with net systolic and diastolic BP reductions of 1.6 and 2.0 mm Hg, respectively^34^. Overall data are insufficient to recommend an increased intake of fibre alone as a means to lower BP. Similarly, high fruit and vegetable intake have been reported to lower BP in small trials but no large study has demonstrated BP lowering effects independent to overall dietary changes^16^,^21^.

### Dietary factors of limited or uncertain effects

Many dietary factors have been evaluated for influence on hypertension control. These include dietary fish oil supplementation, fats other than omega-3 fatty acids, calcium, magnesium, carbohydrates and proteins. Although there are studies supporting their role in BP control, robust scientific evidence from randomized trials is lacking^21^. These factors should, therefore, be considered as of limited or no major importance.

### Comprehensive diet management

Multiple dietary factors are involved in hypertension and it is reasonable that a comprehensive dietary management would be the best approach to manage hypertension and to reduce overall cardiovascular risk.

### DASH and related dietary patterns

Dietary Approaches to Stop Hypertension (DASH) was a program by the National Institutes of Health, USA^16^,^22^,^35^. This series of three large controlled trials tested the effects of dietary patterns on BP. The first trial was a randomized feeding study that compared 3 dietary patterns^16^. Of the 3 diets studied, the most effective diet, now called the DASH diet, emphasized fruits, vegetables, and low-fat dairy products; included whole grains, poultry, fish and nuts; and was low in fats, red meat, sweets, and sugar-containing beverages. Accordingly, it was rich in potassium, magnesium, calcium, and fiber and was low in total fat, saturated fat, and cholesterol; it also was slightly high in protein. It is likely that several aspects of the diet, rather than just one nutrient or food, reduced BP. Among all participants, the DASH diet significantly lowered mean systolic BP by 5.5 mm Hg and mean diastolic BP by 3.0 mm Hg. A second diet, which emphasized just fruits and vegetables also significantly reduced BP but to about half of the effect of the DASH diet^22^. The effects of DASH diet were significantly greater in the black participants (systolic and diastolic BP reductions of 6.9 and 3.7 mm Hg) than in whites (3.3 and 2.4 mm Hg)^35^. The effects in hypertensive individuals (systolic and diastolic BP reductions of 11.6 and 5.3 mm Hg) were striking and were significantly greater than the corresponding effects in non-hypertensive individuals (3.5 and 2.2 mm Hg).

The OmniHeart trial^36^ compared the effects of 3 healthy dietary patterns: a diet rich in carbohydrate (58% of total calories), a second diet rich in protein (about half from plant sources), and a third diet rich in unsaturated fat (predominantly monounsaturated fat). Similar to the DASH diet, each of the OmniHeart diets was low in saturated fat and cholesterol and rich in fruit, vegetables, fiber, potassium, and other minerals. Substituting some of the carbohydrate (10% of total calories) with either protein (about half from plant sources) or unsaturated fat (mostly monounsaturated fat) further lowered BP. The DASH diet and the diets studied in the OmniHeart trial are safe and broadly applicable to the general population. However, because of their relatively high potassium and phosphorus content (in all diets) and high protein content (in the DASH diet and the protein-rich diet in OmniHeart), these diets are not recommended in persons with chronic kidney disease.

## The Indian vegetarian diet

Vegetarian diets have been associated with low BP. In industrialized countries individuals who consume a vegetarian diet have markedly lower BP than do nonvegetarians^37^.Vegetarians also experience a lower age-related rise in BP. Some of the lowest BP observed in developed countries are documented in strict vegetarians. Several aspects of a vegetarian lifestyle might lower BP, including nondietary factors (*e.g*., physical activity), established dietary risk factors (*e.g*., low weight, high potassium, and low-to-moderate alcohol intake), and other aspects of vegetarian diets (e.g., high fibre, no meat). Limited trial evidence, indicates that non-dietary factors and established dietary risk factors are not fully responsible for the BP-lowering effects of vegetarian diets and that some other aspects of vegetarian diets lower BP.

In India, diets in many rural and urban populations are predominantly vegetarian^38^,^39^. BP levels are also lower in these subjects^15^. Large studies that have evaluated comprehensive dietary patterns with BP levels are not available from India. Chhajer *et al*^40^ have modified the Ornish diet^41^ into low-fat Indian vegetarian diet and reported significant reductions in multiple cardiovascular risk factors including BP^42^. Importance of comprehensive dietary change in prevention and management of high BP among Indians has been highlighted^28^,^29^,^43^. Larger controlled interventional studies are needed to confirm superiority of a particular type of Indian vegetarian diet over other similar diets as in India many “healthy” diets are prevalent^38^.

## Weight management

A substantial and consistent body of evidence from observational studies and clinical trials shows that weight is directly associated with BP^13^. The importance of this relationship is reinforced by the high and increasing prevalence of overweight and obesity throughout the world as well as India^44^. Approximately 30-35 per cent or urban Indian adults have a body mass index (BMI) ≥ 25 kg/m^2^ and, therefore, are classified as either overweight or obese^45^ and there is a significant correlation of increasing body weight with hypertension^46^.

With rare exception, clinical trials have documented that weight loss lowers BP. Importantly, reductions in BP occur before, and without, attainment of a desirable body weight. In one meta-analysis that aggregated results across 25 trials, mean systolic and diastolic BP reductions from an average weight loss of 5.1 kg were 4.4 and 3.6 mm Hg, respectively^47^. Additional trials have documented that modest weight loss, with or without sodium reduction, can prevent hypertension by about 20 per cent among overweight, prehypertensive individuals and can facilitate medication step-down and drug withdrawal^18^–^21^. Thus, available evidence strongly supports weight reduction- attainment of a BMI <25 kg/m^2^ or even <23 kg/m^2^ in Indians- as effective approach to prevent and treat hypertension. More importantly, in view of the well-recognized difficulties of sustaining weight loss, efforts to prevent weight gain among those who have normal body weight are critically important. Central or truncal obesity is also a major hypertension risk factor in Indians^48^. Maintaining a healthy body weight (body mass index of 18.5 kg/m^2^ to 24.9 kg/m^2^, preferably < 23.0 kg/m^2^) and waist circumference (<90 cm in men and < 80 cm in women) is therefore recommended.

## Smoking and tobacco intake

Smoking as a hypertension risk factor is not well defined. In western countries epidemiological studies have reported that smokers tend to have lower BP than non-smokers^49^. This is partly accounted for by the fact that smokers tend to be less obese, effect of white coat hypertension is less pronounced in these subjects and usually BP is recorded after abstaining. Ambulatory BP measurements, however, show that the BP of smokers tends to be greater than non-smokers. The effect of smoking on hypertension in less obese subjects of developing countries has not been well studied^50^. In a predominantly bidi smoking population in India we reported that mean systolic BP was significantly greater among men who smoked (rural 127.4 ± 14 vs 125.9 ± 14 mm Hg; urban 126.9 ± 16 vs 123.7 ± 16 mm Hg, *P*<0.01)^51^. The relative risk of hypertension (95% CI) in rural males among mild smokers (1.30, CI 1.00-1.69), moderate smokers (1.39, CI 1.16-1.66) and heavy smokers (1.55, CI 1.03-2.33) was significant. Similar trends were observed in urban men^51^. Many other epidemiological studies from India have reported significant association of smoking and hypertension^50^. Multiple pathophysiological mechanisms explaining smoking with high BP have been postulated^49^. Smoking is one of the more important cardiovascular risk factor in India^52^ and smoking cessation and tobacco control must be an important initial strategy to reduce hypertension as well as overall cardiovascular risk.

## Physical activity

Several large epidemiological studies have reported an inverse relationship between BP and physical activity^13^. Longitudinal intervention studies are more appropriate for assessing the effects of physical activity. In a meta-analysis of randomized controlled trials it was reported that aerobic exercise was associated with a significant reduction in mean systolic (-3.84 mm Hg) as well as diastolic BP (-2.58 mm Hg)^53^. This reduction was observed in hypertensive as well as normotensive individuals and in normal weight as well as overweight participants. On the other hand in another meta-analysis involving 72 trials with 105 study groups, significant reduction in daytime and ambulatory BP was observed, more in hypertensive groups (-6.9/-4.9 mm Hg) than in other groups (-1.9/-1.6, *P*<0.01)^54^. The reduction was associated with decrease in systemic vascular resistance, plasma norepinephrine, plasma rennin activity, body weight, waist size, per cent body fat and insulin resistance. Usefulness of dynamic and static resistance training in BP management is controversial. Regular moderate physical activity is recommended by all the guidelines for BP management^13^ and at least 30 min of moderate physical activity on all days of the week is suggested.

## Yoga and stress management

The role of yogic practices in BP management is controversial. Yogic practices have been reported to reduce BP and multiple cardiovascular risk factors in many studies from India^55^. From UK, Patel *et al*^56^ reported long-term benefit of yoga in reducing coronary risk but a randomized trial of relaxation therapy and meditation in the Netherlands failed to show any benefit on ambulatory BP^57^. In a meta-analysis of lifestyle interventions to reduce raised BP data from 105 trials were included^58^. Robust statistically significant benefits were observed for improved diet, aerobic exercise, alcohol and sodium restriction and fish oil supplements with BP reductions of 5.0/4.6 mm Hg. Relaxation significantly reduced BP only when compared with non-intervention controls and the authors did not recommend this form of therapy for BP control. The American Seventh Joint National Committee (JNC-7) report or the European Society of Hypertension guidelines do not recommend stress management and yoga for hypertension control due to lack of evidence^13^,^59^.

## PHARMACOTHERAPY

Whilst non-pharmacological therapies show modest reductions in BP, all too often these treatments are not adopted and, even if they are, not sustained. Furthermore, in many people the BP remains high despite appropriate non-pharmacological measures especially if the initial BP at diagnosis was high. Under these circumstances pharmacological approaches must be considered. The questions are *(i)* when to treat and *(ii)* with which drug?

### When to initiate therapy?

High BP can be viewed as a *sign* to monitor the patients’ clinical status; or a *risk factor* for atherosclerotic cardiovascular disease or as a *disease* and major contributor to death from cardiac, cerebrovascular, renal or peripheral vascular disease. Currently hypertension is defined as BP equal to or greater then 140/90 mm Hg based on the average of two or more correct BP measurement taken during two or more contacts with health care provider^13^. Higher the BP greater the risk of cardiovascular disease^10^ and, therefore, the JNC-7 defined BP of 120-139/80-89 mm Hg as pre-hypertension^13^. This new category of pre-hypertension, was introduced to emphasize that persons whose BP is >120/80 mm Hg are likely to progress to definite hypertension. It was also hoped that health care providers will encourage persons with BP in pre-hypertension range to begin non-pharmacological lifestyle modifications. The recommendations are that persons with pre-hypertension be treated and evaluated about every month until the BP goal is reached and then every 3-6 months thereafter. Persons with higher level of BP or with complications/end organ damage may need to be evaluated more frequently at regular intervals. Targets of control have been specified for different groups of patients (Table II). It has been recommended that pharmacological therapy should be initiated early if the targets are not achieved by lifestyle changes alone^13^.

**Table II:** Suggested targets for blood pressure control in various co-morbidity groups among adults with hypertension

Sub-group	Target systolic BP mm Hg	Target diastolic BP mm Hg
Usual care uncomplicated hypertension	<140	<90
Diabetes	<130	<80
Coronary heart disease	<130, preferably <120	<80
Chronic renal disease	<130	<80
Congestive heart failure	<120, preferably <110	<80 or <75
Isolated systolic hypertension	<140	-

*Source*: Adapted from Ref 13, 59, 66, 70 and 78

Another aspect of management is the time of the day to administer an antihypertensive medication. Attributes of cardiovascular system are characterized by predictable changes during 24 h cycle^60^; 24 h BP pattern are linked to progressive injury to target organs as well as triggering of cardiac and cerebrovascular events^61^,^62^. Nocturnal hypertension which is characterized by loss of or even reversal of the expected 10-20 per cent sleep-time BP decline, increases the risk of end-organ injury particularly to the heart, brain and kidney^63^. Normalization of the circadian BP rhythm is considered to be an important clinical goal of pharmacotherapy. Thus, treating hypertensive non-dippers involves morning ingestion of medications that exert full 24 h coverage. An evening dosing of appropriate medication could reduce abnormally high BP or convert the disturbed 24 h BP profile to normal dipper pattern and a lower cardiovascular and renal risk. In the HOPE study, nighttime dosing was an important contributor to decreased cardiovascular risk^64^. However, inspite of a great number of published evaluations of antihypertensive agents, the time of day of drug administration has rarely been a specific focus of investigation.

### What initial drug for BP lowering?

The overall goal of treatment in hypertensive patients is to reduce the risk of cardiovascular morbidity and mortality by lowering BP and treating other modifiable risk factors. In general, the goal is to lower BP to below 140/90 mm Hg. In patients with heart failure, diabetes, or renal disease, the goal is to lower BP to below 130/85 mm Hg (Table II). In older patients with isolated systolic hypertension (ISH), the goal is to lower systolic BP to below 140 mm Hg. These goals are achieved through lifestyle modification or drug therapy. However, despite widespread public and professional education regarding the risks of hypertension and the benefits of treatment and despite the ready availability of effective therapies, only 58 per cent of adults with hypertension in the US are receiving treatment and in only 31 per cent is hypertension controlled^13^. In India the situation is worse with very low levels of awareness, treatment and control^3^.

The initial drug management of hypertension is a contentious issue. The JNC-7 report emphasizes on beta-blockers and thiazide diuretics as suitable drugs for first line management^13^. On the other hand, recent studies that have evaluated newer drugs such as ACE inhibitors and CCB molecules report better outcomes using these as compared to the beta-blockers and thiazides^65^. The British National Institute of Clinical Excellence (NICE) guidelines utilize ABCD algorithm for initial pharmacological management of hypertension but has modified these to ACD algorithm in view of changing evidence^66^. Current thinking is to start with an ACE inhibitor in young individuals and CCB in older individuals and step-up the drug therapy until the targets are reached (Table III).

**Table III:** The *ABCDE algorithm* for initial pharmacological management of hypertension

	Young subjects (<55 yr)	Older subjects (>55 yr)
Step I	A or B (if associated sympathetic hyperactivity)	A and/or C
Step 2	Add C or D or both	Add D
Step 3	A or B, C and/or D, add E	A and C, and/or D, add B or E

A, ACE inhibitors/angiotensin receptor blockers; B, beta blockers; C, calcium channel blockers; D, thiazide diuretics; E, extra drugs (centrally acting adrenergic agonists, direct vasodilators, alpha blockers, ganglion blockers, other diuretics, *etc*.). This algorithm has been modified from the British National Institute of Clinical Excellence (NICE) guidelines^66^

One way to improve control could be to start early and utilize combination therapy. The JNC-7 recommends initiation of therapy with combination therapy rather than a single agent if BP is more than 20/10 mm Hg above the treatment goal as in stage II hypertension^13^. A two-drug regimen includes a diuretic appropriate for the level of renal function. An increasing number of antihypertensive combination products are available in a number of dosing especially in India. Although combination products are convenient it is often less expensive to use individual agents and titration of doses of the two agents is easier when the two drugs are prescribed separately. Once BP control is achieved with given doses of two agents, switching to the same therapy in combination is a good option. The advantages and disadvantages of using combination products have been reviewed^67^. Caution is advised when using combination therapy in older persons and diabetic patients, because of the increased risk of precipitous declines in BP or aggravation of orthostatic hypotension. Goal BP may be difficult to achieve in some patients with systolic hypertension, but any reduction is beneficial. Thus, in some patients, a higher systolic goal may be reasonable. In patients who require drugs, lower initial doses should be considered, especially in the presence of orthostatism or co-morbid vascular diseases.

#### Hypertension and diabetes

Patients with diabetes mellitus and hypertension have twice the risk of cardiovascular disease as non-diabetic hypertensive patients. In addition, hypertension increases the risk of diabetic retinopathy and nephropathy^68^. The JNC-7 report as well as American Diabetes Association and the National Kidney Foundation recommends a goal BP of <130/80 mm Hg in hypertensive diabetic patients^13^,^69^,^70^. Many patients with diabetes will require lifestyle modifications and three or more drugs to achieve the BP goals. Meeting these goals may be difficult in some patients. The balance is benefit from lower BP with cost of medication, side effects, and risks associated with the lower goals in some patients. Before initiating drug therapy, it is important to measure BP in the standing position to detect orthostatism, the presence of which may be a clue to autonomic neuropathy and would necessitate a modification to the treatment approach. Numerous studies have shown the effectiveness of ACE inhibitors and ARB in retarding progression of diabetic nephropathy^71^,^72^. For diabetic patients with nephropathy, the American Diabetes Association guidelines recommend ACE inhibitors as initial drugs of choice in type 1 diabetes but ARBs in type 2 diabetes^69^. In some studies, the incidence of cardiac events has been higher in diabetic patients treated with dihydropyridine CCBs, as compared with ACE inhibitors^73^. Beta blockers should be considered in the setting of coronary artery disease, a common co-morbidity in patients with diabetes.

#### Hypertension and kidney disease

Aggressive control of elevated BP can slow progression of renal damage and delay or prevent the development of end-stage disease^70^. The currently recommended goal BP for patients with kidney disease is <130/80 mm Hg. Also, patients with chronic kidney disease are at high risk for cardiovascular morbidity and mortality. Therefore, in addition to elevated BP, other modifiable cardiovascular risk factors require management. ACE inhibitors and ARBs may be more effective than other drugs in slowing progression of proteinuric kidney disease. Serum creatinine concentrations often increase acutely when these drugs are used, so serum creatinine and potassium should be measured within several days of initiating treatment. An increase in creatinine is not a reason to stop the drug unless it is excessive or associated with severe hyperkalaemia. Concomitant use of potassium-sparing diuretics, potassium supplements, or nonsteroidal anti-inflammatory drugs should be avoided. A persistent increase in creatinine with treatment raises the possibility of renal artery stenosis. Most patients with kidney disease will require a diuretic as part of the treatment regimen. If the estimated glomerular filtration rate is < 30 ml/min, thiazide diuretics are usually ineffective, and loop diuretics are required.

### Which drug for reducing overall cardiovascular risk?

The debate continues as to whether the benefits of treating high BP are simply due to the quality of BP control or whether the choice of drug therapy adds to the benefit of BP lowering (beyond BP effect). The experimental basis of the benefits of renin-angiotensin system (RAS) blockade beyond BP lowering is compelling, since angiotensin II is implicated in many pathophysiological changes in the functional and structural disturbances in cardiovascular, cerebrovascular and renal systems^65^. Presently there are data to support claims for drug specific benefits for cardioprotection beyond the effects of BP reduction, but outcome trials have their limitations. They can prove benefit but cannot be considered to provide mechanistic answers of the benefit observed. Large trials such as HOPE^74^ and ONTARGET^75^ have provided some insights. They have shown that there are no real differences between two approaches to RAS blockade but importantly showed that ACEI/ARB combination is not superior to monotherapy alone. The reality in practice is that modern day BP goals do require more than one drug in most of the patients. Since most of the antihypertensive medications have been proven to be safe, the most effective strategy over long term is likely to be one that includes RAS blockade, a diuretic and a CCB^76^.

### Comprehensive risk management

Hypertension is a vascular disease and raised BP is associated with multiple risk factors. It has been shown that as the BP level increases the incidence of cardiometabolic abnormalities increase^77^. Therefore, it is prudent to focus on multiple risk factors while initiating treatment for hypertension. Co-morbid conditions such as dyslipidaemia or diabetes should be addressed^13^,^78^. Low-dose aspirin is considered once BP is controlled^79^. The polypill concept^80^ was developed because it has been realised that more than 90 per cent of individuals have a lifetime risk of developing hypertension^81^ and vascular diseases and are major causes of mortality among hypertensive subjects as well as among all the populations in developed and developing countries^4^,^82^. This implies that to decrease comprehensive vascular risk a combination therapy with multiple BP lowering drugs (thiazide, beta-blocker and ACE inhibitor), aspirin and cholesterol lowering statin is important^80^. Whether this “polypill” should be the initial management of hypertension or should be initiated after comprehensive risk assessment awaits further trials^83^.

In conclusion, hypertension is a major problem in India and the most prevalent chronic disease. Most of the subjects have mild to moderate hypertension and the initial strategies for management involve lifestyle changes focussing on reduction of dietary salt, fat and alcohol and increase in potassium and fruits and vegetables. Weight management and reduction in obesity and truncal obesity, regular physical exercise, tobacco cessation and stress management are important. Pharmacological treatment should be initiated after lifestyle interventions and choice of drug depends on age, the overall cardiovascular risk and co-morbidities. Management should focus on comprehensive risk reduction for better prognosis.

## References

[1] Kearney P, Whelton M, Reynolds K, Muntner P, Whelton PK, He J (2005). Global burden of hypertension: analysis of worldwide data. *Lancet*.

[2] Gupta R, Al-Odat NA, Gupta VP (1996). Hypertension epidemiology in India: meta-analysis of 50 year prevalence rates and blood pressure trends. *J Hum Hypertens*.

[3] Gupta R (2004). Trends in hypertension epidemiology in India. *J Hum Hypertens*.

[4] Murray CJ, Lopez AD (1997). Alternative projections of mortality and disability by cause 1990-2020: Global burden of disease study. *Lancet*.

[5] Kannel WB, Dawber TR, Kagan A, Revotskie N, Stokes J (1961). Factors of risk in the development of coronary heart disease- six year follow-up experience. The Framingham Study. *Ann Intern Med*.

[6] Stamler J, Stamler R, Neaton JD (1993). Blood pressure, systolic and diastolic, and cardiovascular risks: US population data. *Arch Intern Med*.

[7] Vasan RS, Larson MG, Leip EP, Evans JC, O’Donnell CJ, Kannell WB (2001). Impact of high normal blood pressure on the risk of cardiovascular disease. *N Engl J Med*.

[8] Rodgers A, Lawes C, MacMahon S (2000). Reducing the global burden of blood pressure related cardiovascular disease. *J Hypertens*.

[9] Gaziano T, Reddy KS, Paccaud F, Horton S, Chaturvedi V, Jamison DT, Breman JG, Measham AR, Alleyene G, Cleason M, Evans DB, Jha P, Mills A, Musgrove P (2006). Cardiovascular disease. *Disease control priorities in developing world*.

[10] Lewington S, Clarke R, Qizilbash N, Peto R, Collins R (2002). Prospective Studies Collaboration Age-specific relevance of usual blood pressure to vascular mortality: a meta-analysis of individual data for one million adults in 61 prospective studies. *Lancet*.

[11] Turnbull F, Neal B, Algert C, Chalmers J, Chapman N, Cutler J (2005). Blood Pressure Lowering Treatment Trialists’ Collaboration. Effects of different blood pressure-lowering regimens on major cardiovascular events in individuals with and without diabetes mellitus: results of prospectively designed overviews of randomized trials. *Arch Intern Med*.

[12] Kaplan NM (2006). Treatment of hypertension: remaining issues after the Anglo-Scandinavian Cardiac Outcomes Trial. *Hypertension*.

[13] Chobanian AV, Bakris GL, Black HR, Cushman WC, Green LA, Izzo JL (2003). Seventh report of the joint national committee on prevention, detection, evaluation and treatment of high blood pressure. *Hypertension*.

[14] Harrap SB (2003). Where are all the blood pressure genes?. *Lancet*.

[15] Gupta R (1997). Defining hypertension in the Indian population. *Natl Med J India*.

[16] Appel LJ, Moore TJ, Obarzanek E, Vollmer WM, Svetkey LP, Sacks FM (1997). A clinical trial of the effects of dietary patterns on blood pressure: DASH Collaborative Research Group. *N Engl J Med*.

[17] Appel LJ, Champagne CM, Harsha DW, Cooper LS, Obarzanek E, Elper PT (2003). Effects of comprehensive lifestyle modification on blood pressure control: main results of the PREMIER clinical trial. *JAMA*.

[18] (1997). [No authors listed]. Effects of weight loss and sodium reduction intervention on blood pressure and hypertension incidence in overweight people with high-normal blood pressure. The Trials of Hypertension Prevention, Phase II. The Trials of Hypertension Prevention Collaborative Research Group. *Arch Intern Med*.

[19] Stevens VJ, Obarzanek E, Cook NR, Lee IM, Appel LJ, Smith-West D, Trials for the Hypertension Prevention Research Group (2001). Long-term weight loss and changes in blood pressure: results of the Trials of Hypertension Prevention, phase II. *Ann Intern Med*.

[20] Whelton PK, Appel LJ, Espeland MA, Applegate WB, Ettinger WH, Kostis JB (1998). Sodium reduction and weight loss in the treatment of hypertension in older persons: a randomized controlled trial of nonpharmacologic interventions in the elderly (TONE): TONE collaborative research group. *JAMA*.

[21] Appel LJ, Brands MW, Daniels SR, Karanja N, Elmer PJ, Sacks FM; American Heart Association (2006). Dietary approaches to prevent and treat hypertension: a scientific statement from the American Heart Association. *Hypertension*.

[22] Sacks FM, Svetkey LP, Vollmer WM, Appel LJ, Bray GA, Harsha D (2001). DASH-Sodium Collaborative Research Group. Effects on blood pressure of reduced dietary sodium and the Dietary Approaches to Stop Hypertension (DASH) diet. *N Engl J Med*.

[23] Johnson AG, Nguyen TV, Davis D (2001). Blood pressure is linked to salt intake and modulated by the angiotensinogen gene in normotensive and hypertensive elderly subjects. *J Hypertens*.

[24] MacGregor GA, Markandu ND, Sagnella GA, Singer DR, Cappuccio FP (1989). Double-blind study of three sodium intakes and long-term effects of sodium restriction in essential hypertension. *Lancet*.

[25] (1988). [No authors listed]. Intersalt: an international study of electrolyte excretion and blood pressure. Results for 24 hour urinary sodium and potassium excretion. Intersalt Cooperative Research Group. *BMJ*.

[26] Weinberger MH, Miller JZ, Luft FC, Grim CE, Fineberg NS (1986). Definitions and characteristics of sodium sensitivity and blood pressure resistance. *Hypertension*.

[27] Dash SC, Sundaram KR, Swain PK (1994). Blood pressure profile, urinary sodium and body weight in the ‘Oraon’ rural and urban tribal community. *J Assoc Physicians India*.

[28] (2003). Indian Council of Medical Research. *Dietary guidelines for Indians- a manual*.

[29] Misra A, Khurana L (2007). Salt intake and hypertension: walking the tight rope. *J Assoc Physicians India*.

[30] Whelton PK, He J, Cutler JA, Brancati FL, Appel LJ, Follmann D (1997). Effects of oral potassium on blood pressure. Meta-analysis of randomized controlled clinical trials. *JAMA*.

[31] Patki PS, Singh J, Gokhale SV, Bulakh PM, Shrotri DS, Patwardhan S (1990). Efficacy of potassium and magnesium in essential hypertension: a double-blind, placebo controlled, crossover study. *BMJ*.

[32] Klatsky AL, Friedman GD, Siegelaub AB, Gerard MJ (1977). Alcohol consumption and blood pressure Kaiser-Permanente Multiphasic Health Examination data. *N Engl J Med*.

[33] Xin X, He J, Frontini MG, Ogden LG, Motsamai OI, Whelton PK (2001). Effects of alcohol reduction on blood pressure: a meta-analysis of randomized controlled trials. *Hypertension*.

[34] He J, Whelton PK (1999). Effect of dietary fiber and protein intake on blood pressure: a review of epidemiologic evidence. *Clin Exp Hypertens*.

[35] Vollmer WM, Sacks FM, Ard J, Appel LJ, Bray GA, Simons-Morton DG (2001). for the DASH-Sodium Trial Collaborative Research Group. Effects of diet and sodium intake on blood pressure: subgroup analysis of the DASH-sodium trial. *Ann Intern Med*.

[36] Appel LJ, Sacks FM, Carey VJ, Obarzanek E, Swain JF, Miller ER 3 r rd (2005). OmniHeart Collaborative Research Group. Effects of protein, monounsaturated fat, and carbohydrate intake on blood pressure and serum lipids: results of the OmniHeart randomized trial. *JAMA*.

[37] Sacks FM, Kass EH (1988). Low blood pressure in vegetarians: effects of specific foods and nutrients. *Am J Clin Nutr*.

[38] Achaya KT (1994). *Indian food: a historical companion*.

[39] Chadha SL, Gopinath N, KatyaI I, Shekhawat S (1995). Dietary profile of adults in an urban & a rural community. *Indian J Med Res*.

[40] Chhajer B (2008). *Zero oil cook book*.

[41] Ornish D (1990). *Dr Dean Ornish’s program for reversing heart disease*.

[42] Sindhwani V (2004). *Role of dietary modification with zero-oil diet in coronary artery disease patients*. MSc dissertation.

[43] Reddy KS, Katan MB (2004). Diet, nutrition and the prevention of hypertension and cardiovascular diseases. *Public Health Nutr*.

[44] Kim S, Popkin BM (2006). Understanding the epidemiology of overweight and obesity: a real global public health concern. *Int J Epidemiol*.

[45] Gupta R, Deedwania PC, Mohan V, Rao GHR (2006). Obesity and the metabolic syndrome: management issues. *Type 2 diabetes in South Asians: Epidemiology, risk factors and prevention*.

[46] Gupta R, Joshi P, Mohan V, Reddy KS, Yusuf S (2008). Epidemiology and causation of coronary heart disease and stroke in India. *Heart*.

[47] Neter JE, Stam BE, Kok FJ, Grobbee DE, Geleijnse JM (2003). Influence of weight reduction on blood pressure: a meta-analysis of randomized controlled trials. *Hypertension*.

[48] Gupta R, Rastogi P, Sarna M, Gupta VP, Sharma SK, Kothari K (2007). Body-mass index, waist-size, waist-hip ratio and cardiovascular risk factors in urban subjects. *J Assoc Physicians India*.

[49] Beilin LJ, Kaplan NM (2000). The value of lifestyle in the management of hypertension. *Hypertension therapy annual* Martin Dunitz.

[50] Gupta R, Gupta VP, Singh V (2003). Smoking and hypertension: the Indian scenario. *South Asian J Prev Cardiol*.

[51] Gupta R, Agarwal VS, Gupta VP, Soangra MR (1997). Correlation of smoking, blood pressure levels and hypertension prevalence in urban and rural subjects. *J Assoc Physicians India*.

[52] Joshi P, Islam S, Pais P, Reddy S, Dorairaj P, Kazmi K (2007). Risk factors for early myocardial infarction in South Asians compared with individuals in other countries. *JAMA*.

[53] Whelton SP, Chin A, Xin X, He J (2002). Effect of aerobic exercise on blood pressure: a meta-analysis of randomized controlled trials. *Ann Intern Med*.

[54] Fagard RH, Cornelissen VA (2007). Effect of exercise on blood pressure control in hypertensive patients. *Eur J Cardiovasc Prev Rehabil*.

[55] Udupa KN (1985). *Stress and its management by yoga*.

[56] Patel C, Marmot MG, Terry DJ, Carruthers M, Hunt B, Patel M (1985). Trial of relaxation in reducing coronary risk: four year follow up. *Br Med J (Clin Res Ed)*.

[57] Van Montfrans GA, Karemaker JM, Wieling W, Dunning AJ (1990). Relaxation therapy and continuous ambulatory blood pressure in mild hypertension: a controlled study. *BMJ*.

[58] Dickinson HO, Mason JM, Nicolson DJ, Campbell F, Beyer FR, Cook JV (2006). Lifestyle interventions to reduce raised blood pressure: a systematic review of randomized controlled trials. *J Hypertens*.

[59] (2003). European Society of Hypertension - European Society of Cardiology. Guidelines Committee. 2003 European Society of Hypertension-European Society of Cardiology guidelines for the management of arterial hypertension. *J Hypertens*.

[60] Hermida RC, Ayala DE, Fernandez JR, Mojon A, Alonso I, Calvo C (2002). Modeling the circadian variability of ambulatorily monitored blood pressure by multiple-component analysis. *Chronobiol Int*.

[61] Kario K, Pickering TG, Umeda Y, Hoshide S, Hoshide Y, Morinari M (2003). Morning surge in blood pressure as a predictor of silent and clinical cerebrovascular disease in elderly hypertensives; a prospective study. *Circulation*.

[62] Verdecchia P, Porcellati C, Schillaci G, Borgioni C, Ciucci A, Battistelli M (1994). Ambulatory blood pressure. An independent predictor of prognosis in essential hypertension. *Hypertension*.

[63] O’Brien E, Sheridan J, O’Malley K (1988). Dippers and non-dippers. *Lancet*.

[64] Taylor R (2003). Conundrum of the HOPE Study: time of taking ramipril may account for lack of relation between blood pressure and outcome. *BMJ*.

[65] Wiliams B (2006). The year in hypertension. *J Am Coll Cardiol*.

[66] National Institute of Clinical Excellence National Institute of Clinical Excellence. Hypertension: management of hypertension in adults in primary care. www.nice.org.uk/CG034.

[67] Moser M, Pickering T, Sowers JR (2000). Combination drug therapy in the management of hypertension: when, with what, and how?. *J Clin Hypertens*.

[68] Adler AI, Stratton IM, Neil HA, Yudkin JS, Matthews DR, Cull CA (2000). Association of systolic blood pressure with macrovascular and microvascular complications of type 2 diabetes (UKPDS 36): prospective observational study. *BMJ*.

[69] Treatment of hypertension in adults with diabetes (2003). American Diabetes Association. *Diabetes Care*.

[70] (2002). National Kidney Foundation (NKF) Disease Outcome Quality Initiative (K/DOQI) Advisory Board. K/DOQI clinical practice guidelines for chronic kidney disease: evaluation, classification, and stratification. Kidney Disease Outcome Quality Initiative. *Am J Kid Dis*.

[71] Lewis EJ, Hunsicker LG, Bain RP, Rohde RD (1993). The effect of angiotensin-converting-enzyme inhibition on diabetic nephropathy. *N Engl J Med*.

[72] Brenner BM, Cooper ME, de Zeeuw D, Keane WF, Mitch WE, Parving HH (2001). for the RENAAL Study Investigators. Effects of losartan on renal and cardiovascular outcomes in patients with type 2 diabetes and nephropathy. *N Engl J Med*.

[73] Estacio RO, Jeffers BW, Hiatt WR, Biggerstaff SL, Gifford N, Schrier RW (1998). The effect of nisoldipine as compared with enalapril on cardiovascular outcomes in patients with non-insulin-dependent diabetes and hypertension. *N Engl J Med*.

[74] (2000). Heart Outcomes Prevention Evaluation Study Investigators. Effects of an angiotensin-converting-enzyme inhibitor, ramipril, on cardiovascular events in high-risk patients. *N Engl J Med*.

[75] (2008). The ONTARGET Investigators. Telmisartan, ramipril, or both in patients at high risk for vascular events. *N Engl J Med*.

[76] Williams B (2003). Treating hypertension: It is not how you start but where you end that matters. *J Hypertens*.

[77] Sharma AK, Gupta R, Sarna M, Gupta N, Gupta VP (2009). Hypertension, pre-hypertension and cardiovascular risk factors in urban subjects. *South Asian J Prev Cardiol*.

[78] (2001). National Cholesterol Education Program. Executive summary of the third report of the National Cholesterol Education Program (NCEP) Expert Panel on Detection, Evaluation, and Treatment of High blood Cholesterol in Adults (Adult Treatment Panel III). *JAMA*.

[79] Hansson L, Zanchetti A, Caruthers SG, Dahlof B, Elmfeldt D, Julius S (1998). Effects of intensive blood-pressure lowering and low-dose aspirin in patients with hypertension: principal results of the Hypertension Optimal Treatment (HOT) randomized trial. *Lancet*.

[80] Wald N, Law M (2003). A strategy to reduce cardiovascular disease by more than 80%. *BMJ*.

[81] Vasan RS, Beiser A, Seshadri S, Larson MG, Kannel WB, D’Agostino RB (2002). Residual lifetime risk for developing hypertension in middle-aged women and men: The Framingham Heart Study. *JAMA*.

[82] Rodgers RA, Lawes CMM, Gaziano T, Vos T, Jamison DT, Bremen JG, Measham AR, Alleyene G, Cleason M, Evans DB, Jha P, Mills A, Musgrove P (2006). The growing burden of risk from high blood pressure, cholesterol and body weight. *Disease control priorities in developing world*.

[83] Kaplan NM, Opie LH (2006). Controversies in hypertension. *Lancet*.

